# Unsuccessful tuberculosis treatment outcomes across Brazil's geographical landscape before and during the COVID-19 pandemic: are we truly advancing toward the sustainable development/end TB goal?

**DOI:** 10.1186/s40249-024-01184-6

**Published:** 2024-02-18

**Authors:** Reginaldo Bazon Vaz Tavares, Thaís Zamboni Berra, Yan Mathias Alves, Marcela Antunes Paschoal Popolin, Antônio Carlos Vieira Ramos, Ariela Fehr Tártaro, Clara Ferreira de Souza, Ricardo Alexandre Arcêncio

**Affiliations:** 1 Department of Maternal-Infant and Public Health Nursing, University of São Paulo at Ribeirão Preto College of Nursing (USP/RPCN), Avenida Dos Bandeirantes, 3900, Monte Alegre, Ribeirão Preto, São Paulo, Brazil; 2 Federal University of Tocantins, Palmas Campus (FUT), Quadra 109 Norte, Avenida NS 15, Plano Diretor Norte, Palmas, Tocantins Brazil; 3 State University of Minas Gerais, Passos Campus (SUMG), Avenida Juca Stockler, 1130, Bairro Belo Horizonte, Passos, Minas Gerais Brazil

**Keywords:** Tuberculosis, COVID-19, Spatial analysis, Ecological studies

## Abstract

**Background:**

Tuberculosis is one of the most significant infectious diseases for global public health. The reallocation of healthcare resources and the restrictions imposed by the COVID-19 pandemic have hindered access to TB diagnosis and treatment. Increases in unfavorable outcomes of the disease have been observed in Brazil. The objective of this study was to analyze the spatial distribution of unfavorable TB treatment outcomes in Brazil before and during the pandemic.

**Methods:**

An ecological study with spatial analysis was conducted with all 5569 municipalities in Brazil. All reported cases of tuberculosis between January 2010 and December 2021, as well as reported cases of COVID-19 from February 2020 to December 2021, were included. The outcomes studied encompass loss to follow-up, drug-resistant tuberculosis, and death. The Getis Ord GI* technique was employed to assess spatial association, and the Kernel density estimator was used to identify areas with concentrated increases or decreases in outcomes. Bivariate Local Moran's *I* was used to examine the spatial association between outcomes and COVID-19 incidence. The study was approved by the Research Ethics Committee of Ribeirão Preto Nursing School, University of São Paulo.

**Results:**

There were 134,394 cases of loss to follow-up, 10,270 cases of drug resistance, and 37,863 deaths. Clusters of high and low values were identified for all three outcomes, indicating significant changes in the spatial distribution patterns. Increases in concentrations were observed for lost to follow-up cases in the Southeast, while reductions occurred in the Northeast, South, and Midwest. Drug-resistant tuberculosis experienced an increase in the Southern and Southeastern regions and a decrease in the Northeast and South. TB-related deaths showed notable concentrations in the Midwest, Northeast, South, and Southeast. There was an increase in high occurrence clusters for deaths after 2020 and 2021 in the Northeast.

**Conclusions:**

The pandemic has brought additional challenges, emphasizing the importance of enhancing efforts and disease control strategies, prioritizing early identification, treatment adherence, and follow-up. This commitment is vital for achieving the goal of tuberculosis elimination.

## Background

Tuberculosis (TB) is one of the most significant infectious diseases for global public health, and until the emergence of COVID-19, it was the infectious disease responsible for the highest number of deaths annually worldwide [1]. According to estimates from the Stop TB Partnership, the COVID-19 pandemic could lead to an increase of 6.3 million cases and 1.4 million TB-related deaths between 2020 and 2025, considering the 30 countries with the highest burden of the disease [2].

The reallocation of human, material, and financial resources within healthcare services, as well as mobility restrictions, fear of contracting COVID-19, and the socioeconomic impact of the pandemic, have created barriers to accessing TB diagnosis. This has hindered programmatic efforts for active case finding of respiratory symptoms and contact tracing, as well as the monitoring and adherence to treatment [3–5].

It is estimated that in 2021, there were 10.6 million new cases of TB worldwide. This represents an increase compared to 2019, when there were 10 million new cases. For TB-related deaths, the number rose from 1.2 million in 2019 to 1.4 million in 2021 [1].

Brazil is ranked as one of the 30 countries with the highest TB burden in the world, with an incidence of 36.3 cases per 100,000 inhabitants and a mortality rate of 2.3 deaths per 100,000 inhabitants in 2022. Before the pandemic, the proportion of loss to follow-up (LTFU) as a treatment outcome among new cases was 12.6%, rising to 14% in 2021 [6].

On the other hand, the proportion of cases with cure outcome declined sharply, dropping from 73.8% in 2019 to 66.5% in 2021 [drastically below the 85% threshold set by the World Health Organization (WHO)]. For drug-resistant tuberculosis (DR-TB) cases, there was a decrease in detection in the first year of the pandemic, followed by an increase in the two subsequent years, reaching 1104 cases, the highest since 2015 [6] which may be a reflection of the increased LTFU.

In this regard, the impact on services providing assistance to people with TB has been reflected in case detection and resulted in more catastrophic treatment outcomes, compromising the progress made in the last decade in the fight against TB and hindering the achievement of the Global Strategy to End Tuberculosis, which aims to reduce deaths by 95% and incidence by 90% by 2035 [1, 7].

The End TB Strategy is part of the global efforts to control TB and is aligned with the third Sustainable Development Goal (SDG), which includes target 3, establishing the end of the global TB epidemic by 2030 as part of the goal to ensure a healthy life and promote well-being for all, at all ages [1, 7].

In Brazil, TB has a heterogeneous distribution, where the uneven occurrence of treatment outcomes is often associated with socioeconomic disparities, differences in access and quality of healthcare services, and varying population densities among the country's regions [8–11].

Such factors have been investigated using geoprocessing techniques, which enable the identification of priority areas for disease control, as well as the examination of the interaction of these factors at an ecological level through tools for spatial association analysis and point density estimation [12–14].

However, the impact of the COVID-19 pandemic on TB rates may have altered the spatial distribution pattern of the disease, considering the vast dimensions of the country, its diverse regional realities, and the increase in unfavorable outcomes. In the literature, there are few studies [15, 16] on unfavorable treatment outcomes, especially in the post-pandemic period. Generally, studies in Brazil have conducted analyses by region or territories, but not at the national level. While there have been many discussions about the detrimental effects of COVID-19 on TB morbidity and mortality, there is a need for more sensitive investigations to assess the before and after effects of the pandemic.

An analysis from a territorial perspective allows the observation of the varying barriers that individuals with TB encounter throughout their health-disease process, such as difficulties in promptly obtaining a diagnosis, access to medication, and/or inadequate adherence to treatment (whether it is due to user-related healthcare service issues, the need for dignified and humane treatment, a user-centered approach, or social stigma) [17].

Thus, the aim of this study was to analyze the spatial distribution of unfavorable TB treatment outcomes in Brazil before and during the COVID-19 pandemic, as well as to examine the spatial association between these outcomes and the incidence of COVID-19.

## Methods

### Study design and location

This is an ecological study [18], utilizing spatial analysis techniques, conducted in all 5569 municipalities of Brazil.

**Fig. 1:**
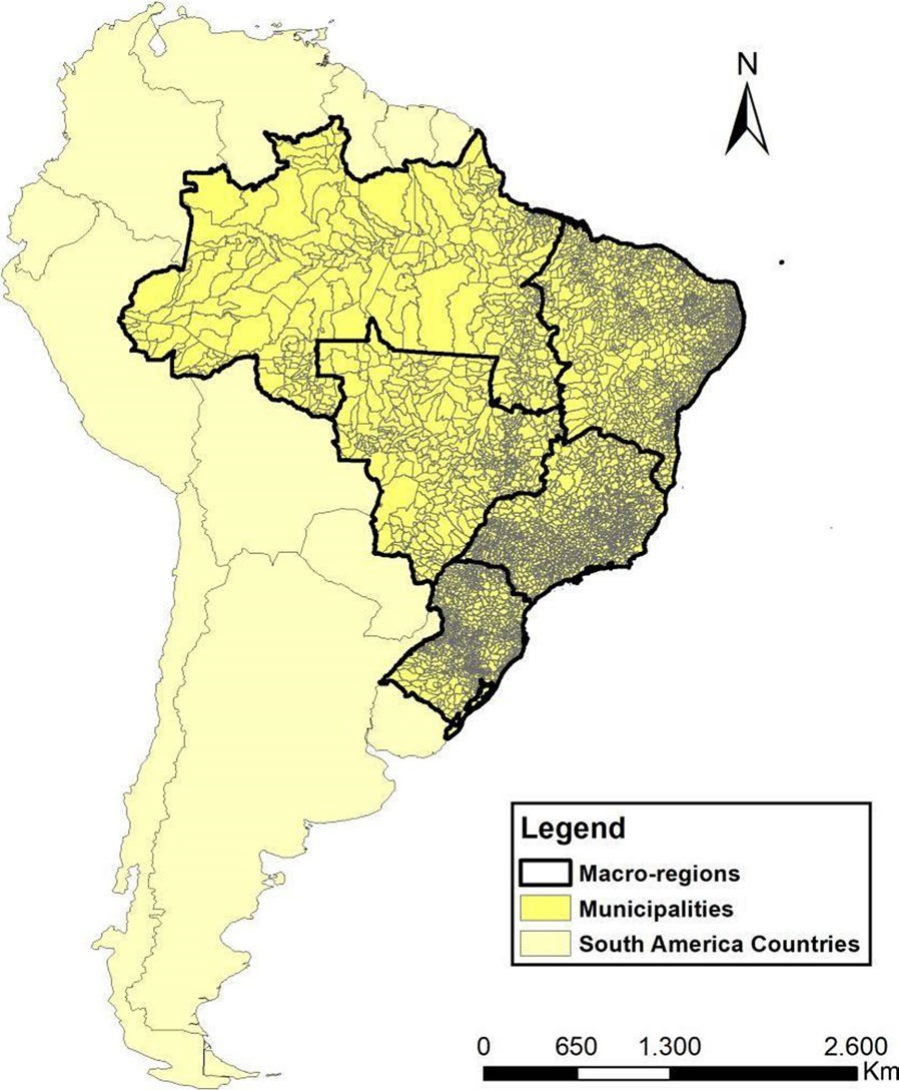
Map of the research location, Brazil

Brazil is the largest country in South America in terms of territory, with an approximate land area of 855,767 square kilometers, divided into five macroregions: North, Northeast, Midwest, South, and Southeast (Fig. 1). It has an estimated population of 207 million people [19].

### Population, information sources and selection criteria

The population consisted of TB cases reported between January 2010 and December 2021, as well as all COVID-19 cases reported between February 2020 and December 2021 in Brazil. The unfavorable outcomes considered were: LTFU, DR-TB, and death.

The treatment outcomes were based on definitions provided by the Brazilian Ministry of Health definitions. LTFU is when a patient used medication for 30 days or more and discontinued the treatment for 30 consecutive days or more. In cases of directly observed treatment (DOT), the 30-day period is counted from the last medication dose [6, 20]. Recently, the Brazilian Ministry of Health changed the terminology for this outcome, replacing “treatment abandonment” (“abandono de tratamento”) with “lost to follow-up” (“interrupção de tratamento” or “perda de seguimento”), which we will adopt here to align with the guidelines for using nonstigmatizing language [6, 21].

DR-TB is characterized by resistance to at least one of the first-line (most effective) medications used to treat the disease (usually isoniazid or rifampicin). It is important to highlight that there are two ways that can lead to infection and illness from DR-TB: primary infection and acquired infection [1, 20].

Acquired resistance results from inadequate, incomplete treatments, with inappropriate drugs or dosage, resulting in *Mycobacterium tuberculosis* strains resistant to first-line medications; while the primary infection occurs through the transmission and infection of a strain of *M. tuberculosis* that is already resistant to medications. When DR-TB is undiagnosed, untreated or mistreated, in addition to making new strain mutations susceptible, it increases the risk of exposure and new infections of already DR-TB [1, 20].

We emphasize that, for the present study, only cases that had DR-TB (acquired resistance) as a treatment outcome were considered, that is, cases of primary resistance were not considered.

Death resulting from tuberculosis is defined as occurring when the disease is the underlying cause, meaning that TB is the condition initiating the chain of pathological events that directly lead to death [20].

The TB data were obtained from the Department of Informatics of the Unified Health System (DATASUS), and the COVID-19 data were extracted from the Coronavirus Panel, both collected in March 2023. Data from 2022 were not used, as it still shows significant incompleteness due to undergoing verification process before being made available, which could potentially impact the quality of the presented results. Population data were sourced from the 2010 Census of the Brazilian Institute of Geography and Statistics (IBGE) [19].

### Data analysis

First, annual incidence rates were calculated and then aggregated for the three time periods analyzed. The pre-COVID-19 pandemic period was defined from January 2010 to December 2019, to examine the historical series of TB and its distribution pattern over time. The pandemic period was defined from February 2020 (when the first case of COVID-19 was recorded in Brazil) to December 2021.

To calculate the incidence rate, the annual number of TB cases was used as the numerator, and the population of each municipality as the denominator, multiplied by the constant 100,000. TB cases were stratified by outcome, and the proportions of LTFU, DR-TB, and deaths were calculated for each Brazilian municipality, following the predefined periods. The numerator was the number of treatment outcomes, and the denominator was the total number of cases multiplied by the constant 100, to obtain the proportion of cases by outcome. The calculations were performed using Microsoft Office Home and Student 2019 software in Excel (Microsoft Corporation, Redmond, WA, U.S.A.).

Choropleth maps of the proportions by treatment outcome were created for both the pre-pandemic and pandemic period. This was done to examine spatial distribution and potential changes in spatial patterns due to the COVID-19 pandemic. The analysis was conducted using ArcGIS 10.8 software (ESRI, Redlands, California, U.S.A). The cartographic bases used were obtained from the collection provided by IBGE.

To assess spatial association, the Getis Ord GI* technique was employed using the previously calculated rates for the three evaluated outcomes. This was carried out through ArcGIS 10.8 software. Interpretation of this statistic is based on the *Z*-score and significance level (α) values. A positive *Z*-score with statistical evidence indicates a spatial clustering of higher event occurrence (hot spot), whereas a negative *Z*-score with statistical evidence indicates a clustering of lower event occurrence (cold spot). Confidence levels of 90%, 95%, and 99% were adopted [22].

The Kernel density estimator was employed to identify areas with a concentration of increased or decreased rates for the evaluated outcomes. Through statistical smoothing, the estimator generates an intensity surface for visual detection of hotspots, indicating clustering in a spatial distribution on a continuous surface. The point distribution is transformed into a smoothed surface and presented as a continuous map, representing varying levels of case intensity [23].

To apply the tool, a point shapefile was created, where each municipality in Brazil corresponded to a point on the map. Each point was assigned a weight calculated by the difference in rates between before the pandemic (2010–2019) and during the pandemic (2020–2021).

Finally, to assess the presence of spatial association between the analyzed outcomes and COVID-19 rates in the municipalities of Brazil, the technique known as Bivariate Local Moran was employed using GeoDa 1.20 software (GeoDa Center for Spatial Data Science, The University of Chicago, Chicago, Illinois, U.S.A.).

The Local Bivariate Moran's *I* measures the degree of association (positive or negative) between the value of the variable of interest in a specific region and another variable in the same region. This allows for the mapping of statistically significant values, generating a choropleth map according to their classification [24]. In this study, the spatial association between COVID-19 incidence rates and the analyzed TB treatment outcomes, including LTFU, death, or the development of DR-TB, was investigated.

The classification can be as follows: High–High, Low–Low, Low–High, and High–Low. It is important to note that high and low values are classified based on the average values of the variables in neighboring regions [23].

### Ethical considerations

The study was approved by the Research Ethics Committee of the School of Nursing of Ribeirão Preto, University of São Paulo (EERP/USP), under The Certificate of Presentation of Ethical Appreciation (CAAE) number: 67512823.0.0000.5393.

## Results

Between 2010 and 2021, there were 1,054,673 reported cases of TB in Brazil. Regarding outcomes, during the same period, there were 134,394 cases of LTFU (12.74% of the total cases), 10,270 cases of DR-TB (0.97% of the total cases), and 37,863 deaths (3.60% of the total cases). Clusters of high and low values were identified for all three outcomes, indicating significant changes in the spatial distribution patterns.

### Spatial distribution of outcome percentages

**Fig. 2:**
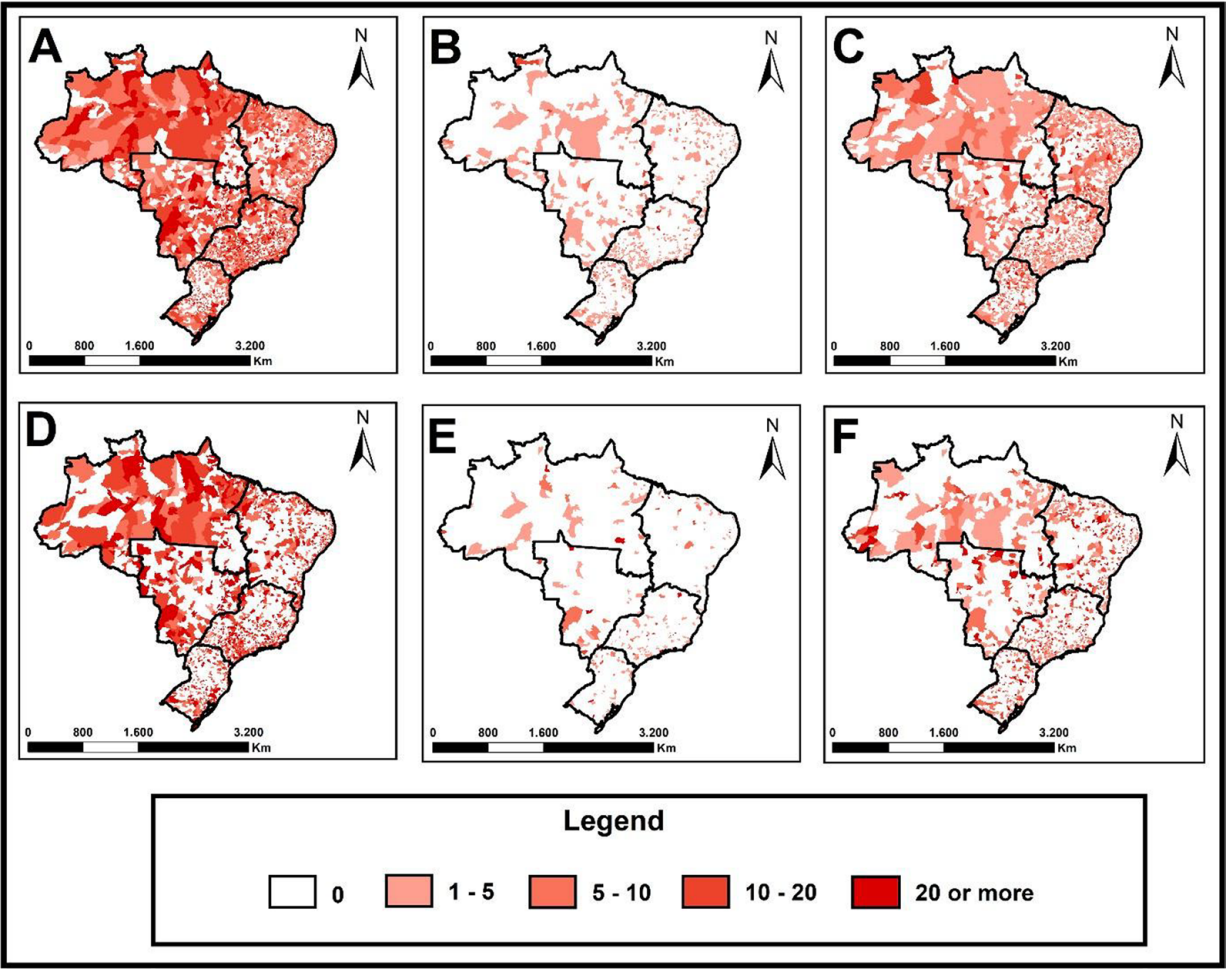
Spatial distribution of rates for unsuccessful outcomes due to tuberculosis, Brazil 2010–2021. **A** Proportion of loss to follow-up for tuberculosis in the pre-pandemic period (Brazil, 2010–2019). **B** Proportion of drug-resistant tuberculosis as an outcome in the prepandemic period (Brazil, 2010–2019). **C** Proportion of deaths as a treatment outcome for tuberculosis in Brazil in the pre-pandemic period (Brazil, 2010–2019). **D** Proportion of loss to follow-up for tuberculosis in the pandemic period (Brazil, 2020–2021). **E** Proportion of drug-resistant tuberculosis as an outcome in the pandemic period (Brazil, 2020–2021). **F** Proportion of deaths as a treatment outcome in the pandemic period (Brazil, 2020–2021)

The spatial distribution of the proportion of TB treatment outcomes can be seen in Fig. 2. It is evident that for the LTFU outcome, municipalities are present and distributed across all regions of the country, with the majority of municipalities having a proportion between 10 and 20%.

It is noticeable that the majority of municipalities have proportions lower than 5% for the development of DR-TB as an outcome, and only 13 municipalities had a proportion greater than 20% in the analyzed period. For the proportion of deaths, most municipalities had values lower than 5%. However, a significant number of municipalities had a proportion between 10 and 20%.

### Spatial association analysis

**Fig. 3:**
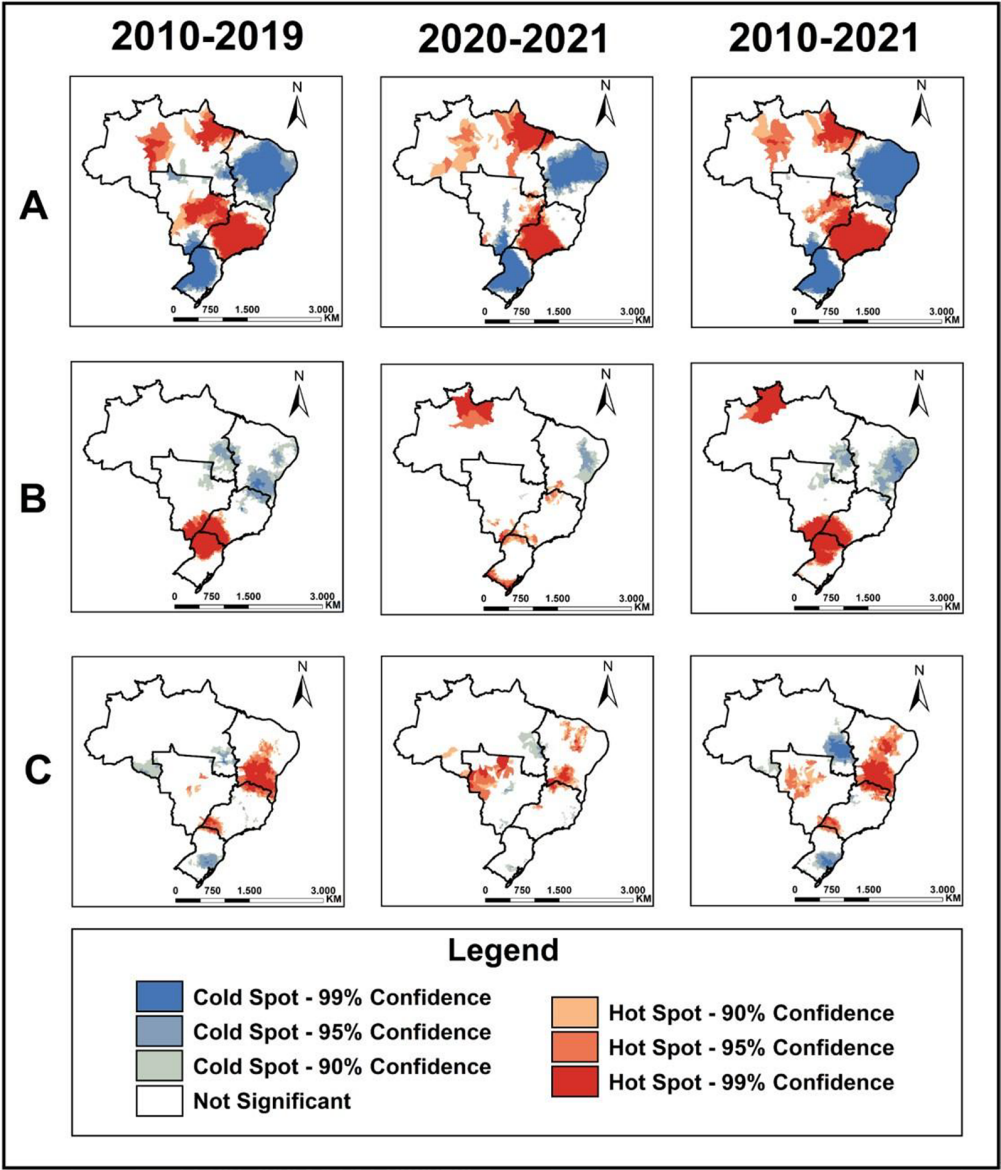
Municipalities with spatial association according to treatment outcomes for tuberculosis, Brazil, 2010–2021. **A** Municipalities with spatial association for loss to follow-up rates in the pre-pandemic period, pandemic period, and complete period, respectively. **B** Municipalities with spatial association for drug resistance rates as a treatment outcome for tuberculosis in the pre-pandemic period, pandemic period, and complete period, respectively. **C** Municipalities with spatial association for death as a treatment outcome for tuberculosis in the pre-pandemic period, pandemic period, and complete period, respectively

Figure 3 shows the results of the local spatial association analysis for the three treatment outcomes during the pre-pandemic period (2010 to 2019), pandemic period (2020 and 2021), and complete period (2010 to 2021). Regarding LTFU (Fig. 3A), similar patterns in cluster formation were observed in all three periods. The Northeast and South regions exhibited clusters of low occurrence, while the Southeast, Midwest, and North regions (specifically Pará, Amapá, and Amazonas) showed clusters of high occurrence.

For cases classified as evolving into DR-TB, the formation of two clusters of high occurrence was observed throughout the periods (Fig. 3B). The cluster located in the far North, concentrated in the state of Roraima, became evident only after 2020–2021. As for deaths (Fig. 3C), a similar pattern of cluster formation with high and low occurrence was observed throughout the entire period. There was an increase in high occurrence clusters after 2020 and 2021 in the Northeast regions, especially in the states of Bahia, Pernambuco, and Ceará, and in the Midwest region, specifically in the state of Mato Grosso.

### Bivariate Local Moran analysis

**Fig. 4:**
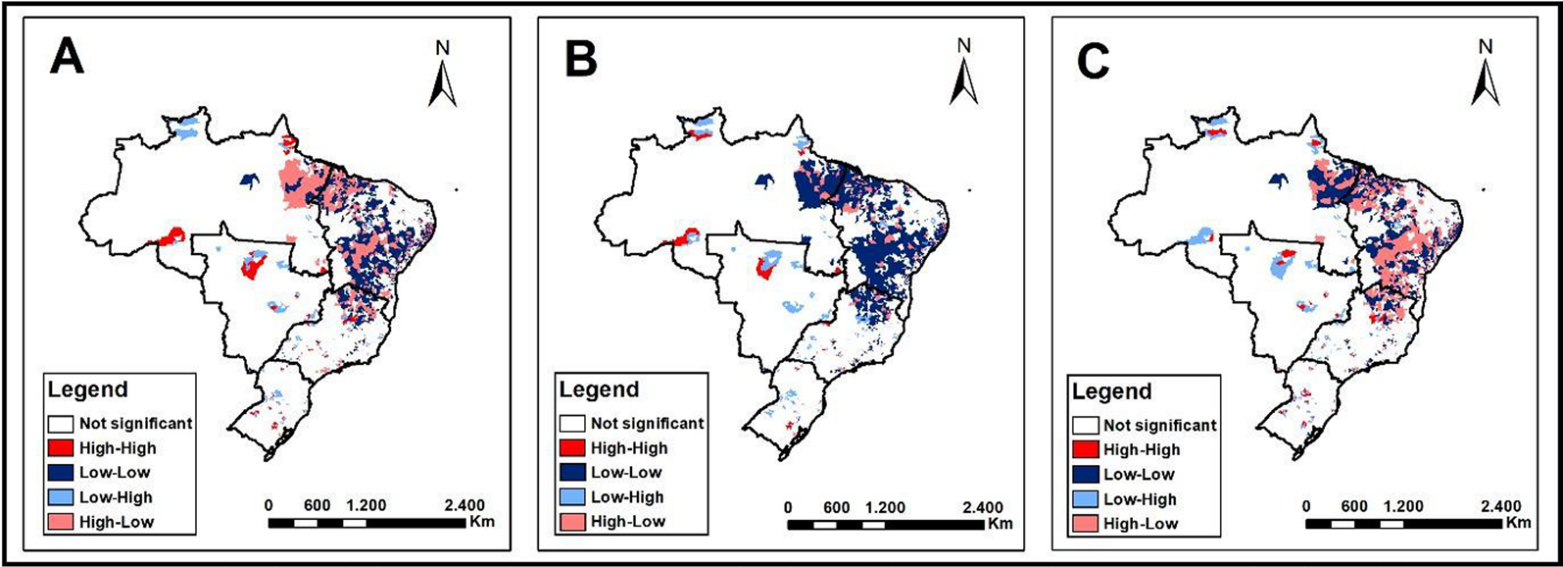
Local bivariate spatial autocorrelation analysis between tuberculosis treatment outcomes and COVID-19 incidence, Brazil, 2010–2021. **A** Application of the local bivariate Moran technique between loss to follow-up in tuberculosis treatment and COVID-19 incidence. **B** Application of the local bivariate Moran technique between drug-resistant tuberculosis and COVID-19 incidence. **C** Application of the local bivariate Moran technique between tuberculosis death and COVID-19 incidence.

The local bivariate analysis allowed us to observe the existence of a spatial association between the proportion of treatment outcomes and the incidence of COVID-19 in municipalities (Fig. 4). It is notable that for LTFU (Fig. 4A), there was a predominance of High–Low areas, meaning municipalities with high proportions of LTFU and low COVID-19 incidence rates, as well as Low–Low areas, representing low proportions of LTFU and low COVID-19 incidence rates. This was particularly prominent in the Northeast region, but it's worth noting the Northern region (Pará) and Southeast region (Minas Gerais) of the country as well.

For DR-TB (Fig. 4B), there was a predominance of Low–Low areas and some High–Low areas, especially in the Northeast region, but also in the Northern region (Pará) and Southeast region (Minas Gerais) of Brazil. Finally, regarding deaths as a treatment outcome for TB, there was a predominance of municipalities classified as High–Low and some Low–Low areas in the same mentioned regions.

### Spatial density analysis

**Fig. 5:**
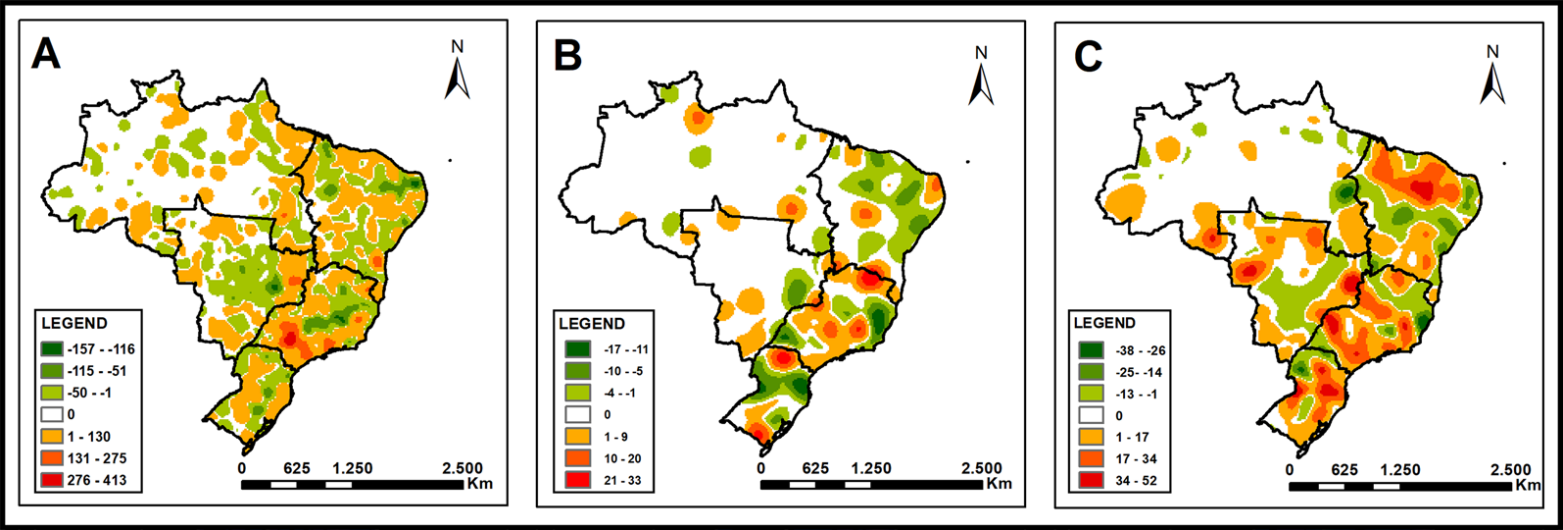
Density of the variation in proportions of unsuccessful tuberculosis treatment outcomes before and during COVID-19. **A** Difference in the proportion of loss to follow-up before (2010–2019) and during (2020–2021) COVID-19. **B** Difference in the proportion of drug-resistant tuberculosis before (2010–2019) and during (2020–2021) COVID-19. **C** Difference in the proportion of deaths before (2010–2019) and during (2020–2021) COVID-19

Figure 5 shows the distribution of density per square kilometer of the difference between the proportion of treatment outcomes before and during the COVID-19 pandemic, highlighting the territories where the new coronavirus may have influenced the increase or decrease in the analyzed outcomes.

For LTFU, the increase was concentrated in municipalities in the Southeast region (São Paulo), while for reduction, there was a concentration in the Southeast (Minas Gerais), Northeast (Bahia), and Midwest (Goiás) regions of the country. For DR-TB, the increase in rates was concentrated in the South (Rio Grande do Sul and Paraná) and Southeast regions, while the reduction was observed in the Northeast and South regions (Santa Catarina). On the other hand, TB-related deaths showed significant concentrations in the Midwest (Mato Grosso and Goiás), Northeast (Bahia and Ceará), South, and primarily, Southeast regions.

## Discussion

The objective of this study was to analyze the spatial distribution of unfavorable TB treatment outcomes in Brazil before and during the COVID-19 pandemic. Additionally, it aimed to assess the spatial association between these outcomes and COVID-19 incidence in Brazil. Furthermore, this study aimed to assess whether advancements are being made in the pursuit of TB elimination.

A decrease in the number of municipalities exhibiting rates of the LTFU was noted as recommended by the WHO, except for the Southeast region, which experienced an increase in LTFU during the pandemic. There was also a reduction in the number of DR-TB cases over the historical series; however, in 2021, the number of cases started to rise again, reaching the highest recorded since 2015 [6]. The study highlighted a significant increase in deaths in certain regions, despite slow progress in the TB situation during the pandemic, exacerbating the situation, as seen in the results of this study.

It was possible to demonstrate that the country is not equally affected by TB, or by COVID-19, as some local systems have shown greater resilience than others. It can also be observed that there are regions with a high risk of DR-TB occurrence, requiring the implementation of strategies to strengthen their system and provide social support for populations affected by poverty and hunger in these areas.

Clusters of high and low values were identified for all three outcomes, indicating significant changes in the spatial distribution patterns of LTFU, progression to DR-TB, and TB-related deaths during the COVID-19 pandemic.

The arrival of the novel coronavirus in Brazil and its evolution into a pandemic has led to socioeconomic repercussions that have exacerbated existing social inequities, particularly affecting those individuals who were already facing greater vulnerabilities, as is the case with people with TB [25, 26]. Thus, the pandemic has intensified the vicious cycle of TB, where the most vulnerable populations end up suffering greater social repercussions from the disease, further aggravating their vulnerabilities [28].

In this context, it is essential to discuss the impacts on TB from a multi-causal perspective, encompassing both biological factors and social determinants that lead to the manifestation of the disease [28]. This calls for action on multiple fronts, including bold social policies and robust healthcare systems, to achieve the agreed upon goals for TB elimination [7]. It is evident, then, that achieving SDG target 3.3 is inseparable from Goals 1 and 10, which addresses the reduction of inequalities and poverty. The implementation of social protection policies aimed at reducing inequality and eliminating extreme poverty are associated with improved TB treatment outcomes [27].

Regarding complications related to the novel coronavirus, coinfections have been noteworthy in individuals affected by COVID-19, especially those with severe cases, including fungal, viral, and bacterial infections, with a particular emphasis on TB [29, 30]. Previous studies [29–31] have demonstrated that the weakened immune state that renders individuals more susceptible to TB can also increase susceptibility to coronavirus infection.

TB and COVID-19, two diseases with distinct histories, have notable similarities, such as their airborne transmission, the presence of key symptoms such as cough and fever, and their high potential to cause structural pulmonary sequelae, in addition to being affected by social stigma [31]. Currently, the COVID-19 pandemic has been identified as one of the main obstacles to the effectiveness of TB control measures on a global scale over the past 2 years [32, 34].

The spatial association analysis of LTFU revealed a similar clustering pattern throughout the entire period in the North, Southeast, and Midwest regions. However, in the comparison between periods, it was observed that during the pandemic there was a decrease in the number of municipalities with a LTFU proportion recommended by the WHO, which is less than 5%, across the entire country.

It is also worth noting that the increase in LTFU proportions during the pandemic was concentrated in the Southeast region. In a historical series up until 2018, municipalities in the Southeast region had the highest average LTFU rate in the country. Additionally, it also concentrated the highest proportions of LTFU, as well as high-risk clusters [35].

The Southeast region is characterized as the country's largest economic hub, which generates a high flow of people through its municipalities, coupled with a high population density [35]. The intense economic activity in the region leads to rapid transformations in its socio-spatial organization, potentially creating contradictory territorial arrangements between areas with full access to urban infrastructure and peripheral regions with precarious housing conditions and limited access to services [36, 37]. Additionally, the average Gini coefficient, a measure of income inequality among the states in the region, is 0.523, highlighting a significant inequality in these communities [38].

Therefore, social inequalities linked to difficulties in accessing healthcare services diminish the quality of life and compromise the population's health. This can be a contributing factor to LTFU in the region, considering that insufficient income to cover transportation costs and challenges in accessing healthcare services are associated with non-adherence to TB treatment [39–41].

Furthermore, it is argued that the high population density of the region poses a continuous challenge for healthcare services, given the need for treatment monitoring and the implementation of DOT, which requires the availability of human resources. This situation may have been exacerbated by the reassignment of professionals during the pandemic [34].

The Southeast region recorded the first cases of COVID-19 in the country and was responsible for the majority of cases [25, 42]. In this context, the magnitude of the pandemic in the region may have impacted healthcare services more intensely than in other regions, leading to the interruption of routine actions and losses in follow-up, especially in Primary Health Care, which is primarily responsible for providing care for chronic health conditions such as TB [34, 43, 44].

The implementation of social distancing and isolation measures, coupled with the fear of contracting COVID-19, may also have contributed to people avoiding visits to healthcare facilities for medication pickup and follow-up, leading to LTFU [5].

These factors, along with other challenges related to TB treatment adherence such as the long treatment duration and adverse effects of medications, may explain the more pronounced increase in LTFU rates in this region [41]. Additionally, it was also observed that the clusters of low occurrence, identified in the Northeast and South regions, remained similar in all three periods analyzed, although it was noted that the rates of LTFU increased among these municipalities.

For DR-TB as treatment outcome, a significant spatial pattern alteration was observed with the formation of a cluster of high rates in the North region and another in the far South of the country during the COVID-19 pandemic. The number of municipalities with a proportion greater than 5% for this outcome decreased when comparing the pre-pandemic and pandemic periods. It is possible to observe that the concentrations of increased and decreased cases showed a heterogeneous distribution in the country.

In the first year of the pandemic, there was a significant reduction in the number of DR-TB as treatment outcome. However, in 2021, the number of cases started to rise again and reached the highest number recorded since 2015 [6]. Therefore, the decrease in the proportions of this outcome observed in some areas may have occurred due to underreporting during this period. This is because the impact of the pandemic on healthcare services in the country led to a reduction in the use of the Rapid Molecular Test for TB diagnosis, a diagnostic method that allows for the assessment of rifampicin-resistant strains [45, 46], which is the main form of resistance observed in the country [6]. It is worth noting that municipalities equipped with this technology are concentrated in the South, Southeast, and Northeast coastal regions [47], which may have contributed to the more evident variation in distribution in these regions.

It is worth noting that the Northeast region remained a cluster of low rates for DR-TB as treatment outcome, as previously observed before the pandemic. Therefore, it is valid to consider the possibility that cases may not be diagnosed and/or reported, which justifies the result. However, it is important to approach these regions with caution and view them as warning signs so that municipal managers can identify situations where reported data may differ from the actual behavior of the disease in the municipalities [48, 49].

Drug resistance poses a significant challenge to TB control, especially in the context of Brazil, a vast and diverse country. The occurrence of drug resistance cases is associated with population density and socioeconomic conditions. However, detecting the disease depends on an appropriate diagnostic network that provides sensitivity tests and cultures of the mycobacteria [50].

Additionally, the clustering of DR-TB is also influenced by the transmission of resistant strains in the community or by the development of drug resistance, mainly due to treatment failures, as considered in the present study [51]. Thus, the spatial distribution of these cases is influenced by the interaction of various factors in communities, resulting in its heterogeneous and complex pattern in the country.

It is important to highlight that a person who undergoes LTFU once is associated with a greater likelihood of not completing retreatment and is more susceptible to developing drug resistance. Therefore, it becomes more challenging to control the TB transmission chain if the number of drug-resistant M. tuberculosis strains increases.

When we address topics such as LTFU or the development of drug resistance, it is crucial to emphasize the situation of indigenous people, where the majority reside in the border regions of Brazil and, as well as the migrant population, have a significant influence on maintaining these rates. TB rates are notably higher among indigenous peoples and migrant populations throughout Latin America, especially in Brazilian Amazonian groups, with an incidence that is up to 20 times higher than that observed in the general population of Brazil [52]. Studies [52, 53] indicate that indigenous people are disproportionately affected by TB compared to other races/ethnicities, but the reasons for these disparities have not yet been fully understood.

The cultural diversity of indigenous peoples in Brazil, which encompasses more than 300 ethnicities distributed across all states of the country and who speak more than 270 languages, reinforces the nation's multicultural identity. However, this diversity also presents considerable challenges for the creation and implementation of more inclusive and differentiated public policies [52, 54].

Furthermore, we face several challenges in relation to the health of the indigenous population, such as difficulty in accessing communities, the lack of local infrastructure and the scarcity of human and material resources [54]. These factors contribute to the discontinuity of health actions, making it essential to highlight the difficulties associated with the transculturation process. It is essential to raise awareness among teams that work in indigenous communities, seeking to balance the assistance of health professionals with traditional belief systems and healing [52].

In this context, both clusters identified during the pandemic period were located in international border regions of Brazil with Uruguay (South) and Venezuela (North). Other studies have also shown the formation of clusters of DR-TB in border regions, in Mongolia [55] and Ethiopia [51] indicating that migration is a significant factor for DR-TB control [56].

The pandemic period was marked by restrictions on movement, leading to a significant decline in migrant mobility across these borders [57]. However, in the two preceding years, 2018 and 2019, the country experienced the highest influx of international migration, primarily driven by the entry of Venezuelans through the Northern Region, in the state of Roraima [58], where the most significant cluster was observed during the pandemic period.

In addition, the pandemic restricted the policies of internalization for migrants, causing this large contingent to be stranded in the state, and exacerbating the vulnerability of this population, particularly in terms of limited access to healthcare services [59]. It is worth noting that Venezuela is facing a profound political, economic, and social crisis, and has an estimated incidence of 47 cases per 100,000 inhabitants of TB, and an incidence of two cases per 100,000 inhabitants of DR-TB for 2021, higher than that found in Brazil [60].

The migration profile to the municipalities in the South region cluster is more diverse, with people coming primarily from Uruguay, but also from Senegal, Venezuela, and Haiti. The latter represents the second largest contingent of migrants in the state [61]. Migration is a complex process that has health implications related to precarious transportation and housing conditions, as well as the epidemiological indicators of the country of origin and destination [62].

Considering this, it is possible that the vulnerabilities associated with the migration process played a significant role in the formation of clusters for DR-TB during the pandemic period, highlighting the relevance of this issue for TB control in the post pandemic period.

For deaths, during the pandemic period, a cluster was observed in the Midwest region, and an existing cluster previously identified in the Northeast region expanded. These clusters coincided with the municipalities that experienced the greatest increases in the proportion of deaths, indicating a shift in the spatial distribution of TB related deaths after 2020. It is worth noting that among the analyzed outcomes, TB related deaths exhibited the highest concentrations of increases across all five regions.

The clusters with low rates experienced increases and remained similar during the pandemic, despite the documented rise in proportions of deaths in these municipalities. This growth is concerning as it signifies a setback in the effort to reduce tuberculosis-related deaths in many regions of Brazil, relative to the established target. To achieve a 95% reduction by 2035, it would be necessary to limit the number of deaths from the disease to less than 230 per year [63].

The Central-West region historically presents the lowest burden of TB among the five regions of the country, with incidence and mortality rates below the national average, as well as better disease monitoring indicators [9, 10]. It is the region with the second-largest territorial extension but the smallest population in the country. It consists of four federal units, including three states and the Federal District, with a heterogeneous population distribution, with some urban centers having a high population concentration and vast areas with low demographic density. It is worth noting that the state of Mato Grosso, where most of the municipalities in the cluster were observed, has the lowest population density in the region, with most municipalities in the state are classified as predominantly rural [35, 64].

A time series study [65] in Brazil identified a growing temporal trend in the TB incidence rate from 2010 to 2019 across the country and all its macro-regions. Furthermore, in 2020, the immediate consequence of the COVID-19 pandemic was a reduction in new diagnoses/notifications. These numbers rose again in 2021 as the pandemic was being controlled [65].

One aspect that is important to highlight is that the COVID-19 pandemic brought challenges to several sectors, including epidemiological surveillance and TB Control Programs, in addition to the need to reorganize services previously dedicated to TB control and lack of personal protective equipment and training of health professionals in relation to the differential diagnosis of TB and COVID-19. Furthermore, it is noteworthy that in most regions, states and municipalities, there was a need to manage human resources to act on the front line against COVID-19, which has a direct impact on the operationalization of the DOT and consequently on the actions of active search for respiratory symptoms, also compromised by social distancing [5, 66, 67].

Given this, in 2020 there was a drastic decrease in the number of diagnosed cases and, consequently, in the notification of favorable or unfavorable outcomes. According to the WHO, there were around 1.4 million fewer cases of TB that were not detected or treated in 2020, a 21% reduction compared to 2019 [33]. In Brazil, in 2020 the average number of reported TB cases decreased by around 6.5 cases compared to the period from 2017 to 2019, with the exception of the Northern Region of the country [33].

As limitations of the present investigation, it is important to note that this is an ecological study, which means we must acknowledge the presence of the so-called ecological fallacy. In this type of study, when using aggregate-level data, the results may not directly reflect associations at an individual level, underscoring the need to conduct further research with different designs and methods to draw more precise and reliable conclusions. Additionally, it is relevant to consider the use of secondary data sources, which may lead to incomplete information or typing errors, potentially affecting the results.

Another limitation of the study that should be mentioned is the use of population-based data obtained from the last official demographic census conducted in 2010 to calculate rates. Due to this lag, demographic data may not fully reflect the population reality during the study period, which can potentially impact the analyses conducted.

However, the application of spatial analysis techniques enables an understanding of the disease's behavior over time and the identification of problematic areas in the country regarding TB. These issues may have been exacerbated due to the COVID-19 pandemic. Such information is valuable in assisting health managers in making more informed decisions, facilitating the comprehension of the disease transmission chain, and understanding the context in which the population is situated.

Therefore, identifying critical areas becomes a priority tool in tackling TB. It enables the development of strategies such as active case finding, contact tracing, proper treatment monitoring to prevent drug resistance, ensuring individualized treatment with a multidisciplinary team to meet the patient's needs, and improving treatment adherence. Additionally, controlling symptoms and potential medication side effects and addressing social determinants of health are essential. Utilizing social protection strategies as support for patients and monthly monitoring of diagnosed individuals are crucial components of this approach.

## Conclusions

The issue of TB and its relationship with the COVID-19 pandemic is of utmost importance in the field of public health. Identifying critical areas and understanding how the COVID-19 pandemic may have impacted the rates of unfavorable TB treatment outcomes are crucial for guiding health managers in making strategic and effective decisions, ultimately contributing to the achievement of TB elimination goals.

In this context, the COVID-19 pandemic has brought additional challenges to TB control, underscoring the need to enhance epidemiological surveillance strategies and invest in preventive measures. By prioritizing early case detection, treatment adherence, and continuous monitoring of individuals diagnosed with TB, it will be possible to effectively combat the disease and promote overall public health improvement. This is a vital commitment for the progress of our society and for achieving the goals set in the 2030 Agenda.

## Data Availability

All the data supporting the study findings are within the manuscript. Additional detailed information and raw data will be shared upon request addressed to the corresponding author.

## Abbreviations

CAAE: Certificate of Presentation for Ethical Appreciation
DATASUS: Department of Informatics of the Unified Health System
DOT: Directly observed treatment
DR-TB: Drug-resistant tuberculosis
IBGE: Brazilian Institute of Geography and Statistics
LTFU: Lost to follow-up
SDG: Sustainable Development Goals
TB: Tuberculosis
WHO: World Health Organization
